# Radiologic pleuroparenchymal fibroelastosis-like lesion in connective tissue disease-related interstitial lung disease

**DOI:** 10.1371/journal.pone.0180283

**Published:** 2017-06-30

**Authors:** Yasunori Enomoto, Yutaro Nakamura, Thomas V. Colby, Takeshi Johkoh, Hiromitsu Sumikawa, Koji Nishimoto, Katsuhiro Yoshimura, Sayomi Matsushima, Yoshiyuki Oyama, Hironao Hozumi, Masato Kono, Tomoyuki Fujisawa, Noriyuki Enomoto, Naoki Inui, Toshihide Iwashita, Takafumi Suda

**Affiliations:** 1 Second Division, Department of Internal Medicine, Hamamatsu University School of Medicine, Shizuoka, Japan; 2 Department of Regenerative and Infectious Pathology, Hamamatsu University School of Medicine, Shizuoka, Japan; 3 Department of Laboratory Medicine and Pathology (Emeritus), Mayo Clinic Arizona, Scottsdale, AZ, United States of America; 4 Department of Radiology, Kinki Central Hospital of Mutual Aid Association of Public Teachers, Hyogo, Japan; 5 Department of Radiology, Osaka Medical Center for Cancer and Cardiovascular Diseases, Osaka, Japan; 6 Department of Clinical Pharmacology and Therapeutics, Hamamatsu University School of Medicine, Shizuoka, Japan; Keio University, JAPAN

## Abstract

**Background:**

Radiologic pleuroparenchymal fibroelastosis (PPFE)-like lesion including pulmonary apical cap can be occasionally observed in clinical settings. However, the significance of radiologic PPFE-like lesion is unclear in connective tissue disease (CTD)-related interstitial lung disease (ILD).

**Materials and methods:**

A total of 113 patients with CTD-related ILD were enrolled and assessed for radiologic PPFE-like lesion, which was defined as bilateral, upper lobe, and subpleural dense consolidations with or without pleural thickening on chest high-resolution computed tomography. The clinical, radiologic, and pathologic characteristics were evaluated.

**Results:**

Radiologic PPFE-like lesion was found in 21 patients (19%) and were relatively frequent in those with systemic sclerosis (6/14: 43%) and primary Sjögren's syndrome (4/14: 29%). Patients with PPFE-like lesion were significantly older, had lower body mass index, higher ratio of residual volume to total lung capacity, and higher complication rate of pneumothorax and/or pneumomediastinum than those without. Twelve of the 21 patients were diagnosed radiologically as usual interstitial pneumonia (UIP) or possible UIP pattern. Two of three patients who underwent surgical lung biopsy of the upper lobes showed UIP on histopathology. Another patient was confirmed to have upper lobe PPFE on autopsy. During the clinical course, progression of the radiologic PPFE-like lesions was observed in 13 of 21 patients. Six patients died (mortality rate: 29%) and their PPFE-like lesions were commonly progressive. In the total cohort, our multivariate analysis identified the presence of PPFE-like lesion as a significant risk factor for respiratory death (hazard ratio: 4.10, 95% confidence interval: 1.33–12.65, p = 0.01).

**Conclusion:**

In patients with CTD-related ILD, radiologic PPFE-like lesion, which may present as not only PPFE but also apical cap and upper lobe subpleural fibrosis commonly due to UIP, was not uncommon and was associated with poor prognosis. Clinicians should be cautious with this radiologic finding, particularly when it is progressive.

## Introduction

Pulmonary apical cap is known as a non-morbid finding on chest X-ray or computed tomography (CT). This radiologic term represents an irregular density on the apex of lung that is generally less than 5 mm. The typical pathologic feature is a subpleural fibroelastic scar and is commonly accompanied by thickening of the visceral pleura [1,2]. Changes secondary to other conditions, including infection/post-infectious scarring (e.g., tuberculosis-related changes), post-radiation fibrosis, and lung or pleural neoplasms, can mimic an apical cap. Even when those other diseases or conditions are clinically excluded, an apical cap can be difficult, perhaps somewhat arbitrary, to discriminate from other apical lesions showing subpleural dense consolidation with or without pleural thickening, notably pleuroparenchymal fibroelastosis (PPFE) and fibrosis due to usual interstitial pneumonia (UIP) on upper lobes [3].

Connective tissue diseases (CTDs) are among the important etiologies of interstitial lung disease (ILD). In contrast to idiopathic pulmonary fibrosis (IPF) and idiopathic nonspecific interstitial pneumonia (NSIP), both of which typically show lower lobe-predominant distribution, CTD-related ILD occasionally reveals an upper lobe predominance and, rarely, can coexist with pleural/subpleural disorders, including PPFE [4–6]. Based on this context, we hypothesized that radiologic PPFE-like lesion that mimics or includes an apical cap can be observed in CTD-related ILD; however, its clinical significance is unclear.

In this retrospective study, we investigated the prevalence of PPFE-like lesion on chest high-resolution CT (HRCT) in patients with CTD-related ILD and compared the clinical, radiologic, and pathologic characteristics between patients with and without the finding. Our aim was to evaluate the clinical significance of radiologic PPFE-like lesion in CTD-related ILD.

## Methods

This study was approved by the institutional review board of Hamamatsu University School of Medicine (approval number 15–197). Because of the retrospective nature of this study, written informed consent from the subjects was waived.

### Study population

A retrospective database review of patients with ILD at Hamamatsu University Hospital in Japan between 2001 and 2014 identified 133 patients who were diagnosed with CTD-related ILD. Among them, 20 patients were excluded because their follow-up period was shorter than three months. Subsequently, 113 patients with CTD-related ILD were included in the present study. The diagnosis of ILD was based on chest HRCT findings of reticular abnormalities, ground-glass attenuation, or consolidation and clinical exclusion of other pulmonary diseases, such as infectious pneumonia. The time of ILD diagnosis was based on the first HRCT evaluation. Surgical lung biopsy specimens were available for review in 36 patients. Among them, 30 patients had upper lobe specimens in addition to lower lobe specimens. All patients had been diagnosed with CTD before or at the time of ILD diagnosis. The diagnoses of CTDs were based on accepted criteria [7–12].

### Clinical data

The medical records were retrospectively reviewed to obtain the following clinical data at the time of ILD diagnosis: demographics; smoking history; laboratory data; pulmonary function test results [forced vital capacity (FVC), diffusing capacity of the lung for carbon monoxide (DLCO), and the ratio of residual volume (RV) to total lung capacity (TLC)]; and bronchoalveolar lavage fluid analysis. The patients’ clinical course, including treatment, major respiratory complications, such as acute exacerbation of ILD; respiratory tract infection; lung cancer; and pneumothorax and/or pneumomediastinum, and prognosis, was also recorded. Acute exacerbation of CTD-related ILD was diagnosed, as described previously [13].

### Radiologic evaluation

HRCT scans of the chest at full inspiration were obtained from all 113 patients at the time of ILD diagnosis. All images were reviewed independently by two chest radiologists with 28 and 14 years of experience, respectively, without knowledge of the clinical and pathologic information of the patients.

**Fig 1:**
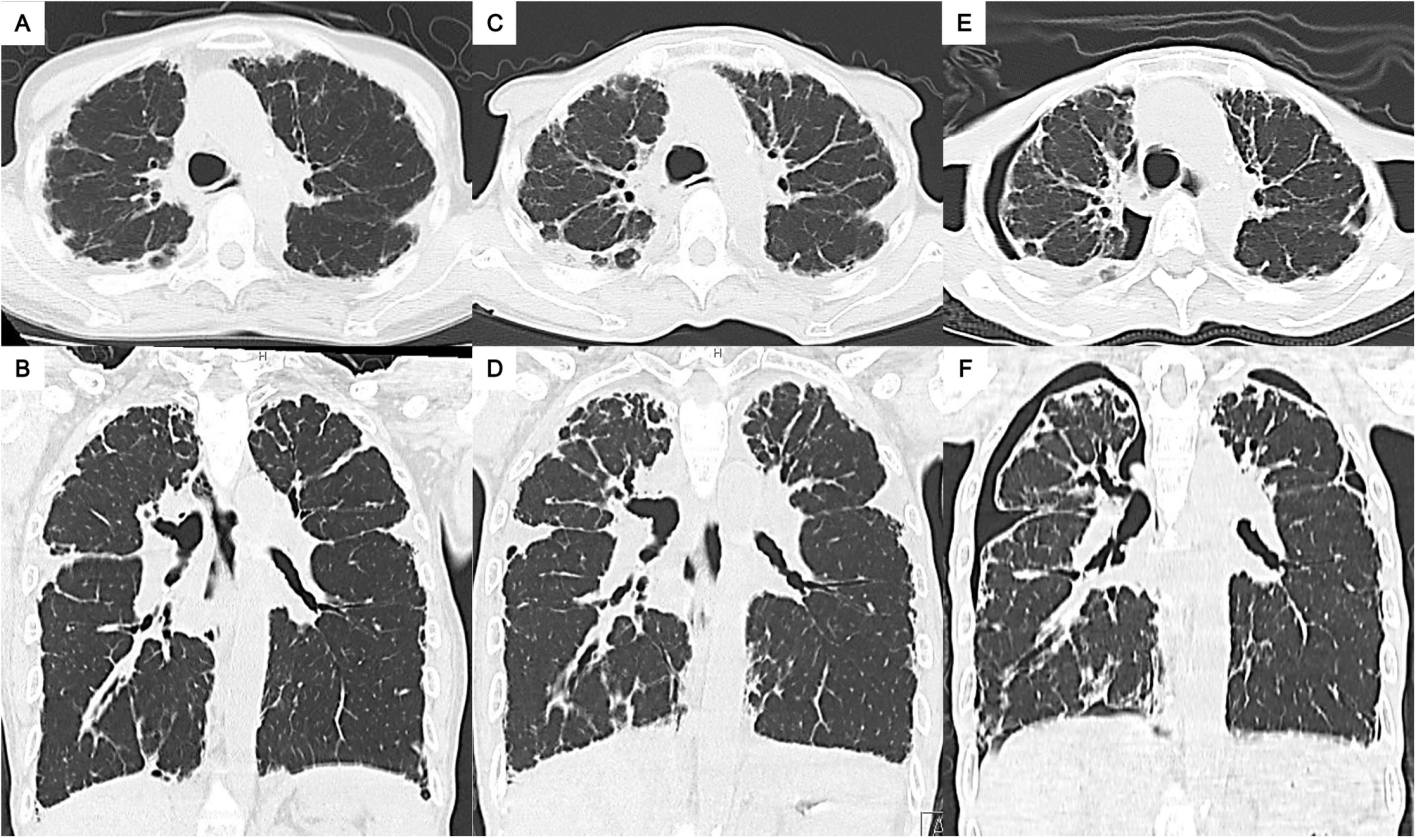
Representative chest computed tomography images of radiologic pleuroparenchymal fibroelastosis (PPFE)-like lesion in a patient with systemic sclerosis and secondary Sjögren's syndrome. Images of patient 18 in S1 Table. (A) and (B): At the time of diagnosis of interstitial lung disease. (C) and (D): One year after the diagnosis. (E) and (F): Two years after the diagnosis. Chronologically, the upper lobe PPFE-like lesion increased; the lung volume, particularly at the upper lobes, seemed to have decreased. Finally, the complication of bilateral upper lobe pneumothorax was found on the background of an emaciated body.

**Fig 2:**
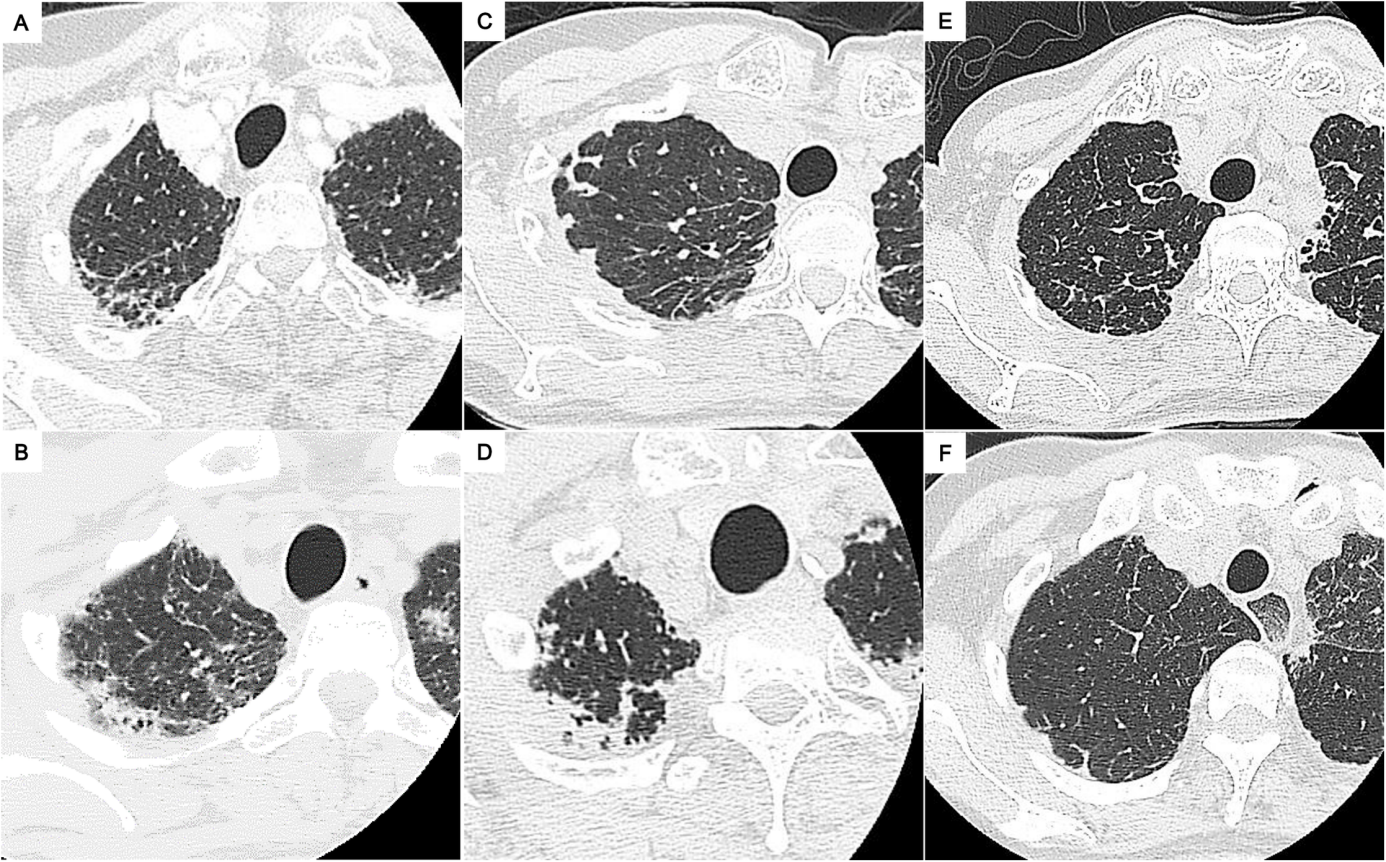
Representative chest computed tomography images of radiologic pleuroparenchymal fibroelastosis (PPFE)-like lesion. Images of the right lung apex in patient 2 (A), patient 9 (B), patient 11 (C), patient 16 (D), patient 17 (E), and patient 20 (F) are shown. The images (E) and (F) reveal minimal cases of PPFE-like lesion we defined. Each patient number is listed in S1 Table.

Radiologic PPFE-like lesion was defined as bilateral, upper lobe, and subpleural dense consolidations with or without pleural thickening on chest HRCT, which corresponds to “consistent with PPFE on radiology” proposed by Reddy et al. [14]. The possibilities of active pulmonary infection and neoplasm were carefully excluded by reviewing the radiologic changes, laboratory data, and clinical course. Representative images are shown in Fig 1 and Fig 2. The extent of reticular abnormalities (reticulation and honeycombing), ground-glass attenuation (in the absence of reticular abnormalities), consolidation, and emphysema were semi-quantitatively scored based on the extent of lung parenchymal involvement: grade 0 (0%), 1 (<25%), 2 (25%–50%), 3 (50%–75%), and 4 (>75%). The cephalocaudal distribution (upper lobe-predominant, lower lobe-predominant, or diffuse) was also evaluated. The HRCT patterns were classified as either UIP, possible UIP, NSIP, NSIP + organizing pneumonia (OP), OP, or unclassifiable based on the criteria mentioned in the 2011 IPF guideline by American Thoracic Society (ATS)/European Respiratory Society (ERS)/Japanese Respiratory Society/Latin American Thoracic Association and in the 2015 research statement for interstitial pneumonia with autoimmune features by the ATS/ERS [15,16]. Disagreements between the two radiologists were resolved by consensus. The simple kappa values were 0.41 for radiologic PPFE-like lesion, 0.35 for cephalocaudal distribution, and 0.27 for ILD patterns. The weighted kappa values for extent of reticular abnormalities, ground-glass attenuation, consolidation, and emphysema were 0.95, 0.97, 0.98, and 0.98, respectively.

### Pathologic evaluation

Histologic sections of lung specimens, from 36 patients by surgical lung biopsy and from 1 patient by autopsy, were stained with hematoxylin–eosin and elastic van Gieson. All slides were reviewed by an experienced lung pathologist (TVC) who was not aware of the clinical and radiologic findings.

For the surgical lung biopsies, the following pathologic findings were semi-quantitatively graded as 0 (absent), 1 (mild), 2 (moderate), or 3 (severe): interstitial fibrosis; fibroblastic foci; lymphoplasmacytic infiltration; lymphoid follicles with germinal center; and emphysema. The presence or absence of a finding of PPFE, defined as upper-zone pleural fibrosis with subjacent intra-alveolar fibrosis accompanied by alveolar septal elastosis (i.e. “definite PPFE on histopathology” proposed by by Reddy et al. [14]), was also evaluated. Subsequently, the pathologic ILD pattern in each patient was determined in accordance with the 2002 classification of idiopathic interstitial pneumonia (IIP) by the ATS/ERS and the 2013 update statement [17,18].

### Statistical analysis

Data were described as number (percentage) or median (interquartile range), unless otherwise stated. Group comparisons were performed using Chi-square test, Fisher’s exact test, or Mann–Whitney U test, as appropriate. Overall survival was defined as the time from the date of ILD diagnosis to the date of censoring or death. Patients were censored if they remained alive until June 30, 2015 or if they dropped out of the follow-ups. The survival curves were generated by the Kaplan–Meier method and were compared by log-rank test. Cox proportional hazard model was used to evaluate the prognostic impact of radiologic PPFE-like lesion, in conjunction with other well-known prognostic factors in ILDs, including age, gender, % predicted FVC, % predicted DLCO, and UIP compatibility on HRCT [19,20]. All statistical analyses were performed using SPSS software version 13.0 (SPSS, USA). A value of p <0.05 was considered to be statistically significant.

## Results

### Baseline characteristics

**Fig 3:**
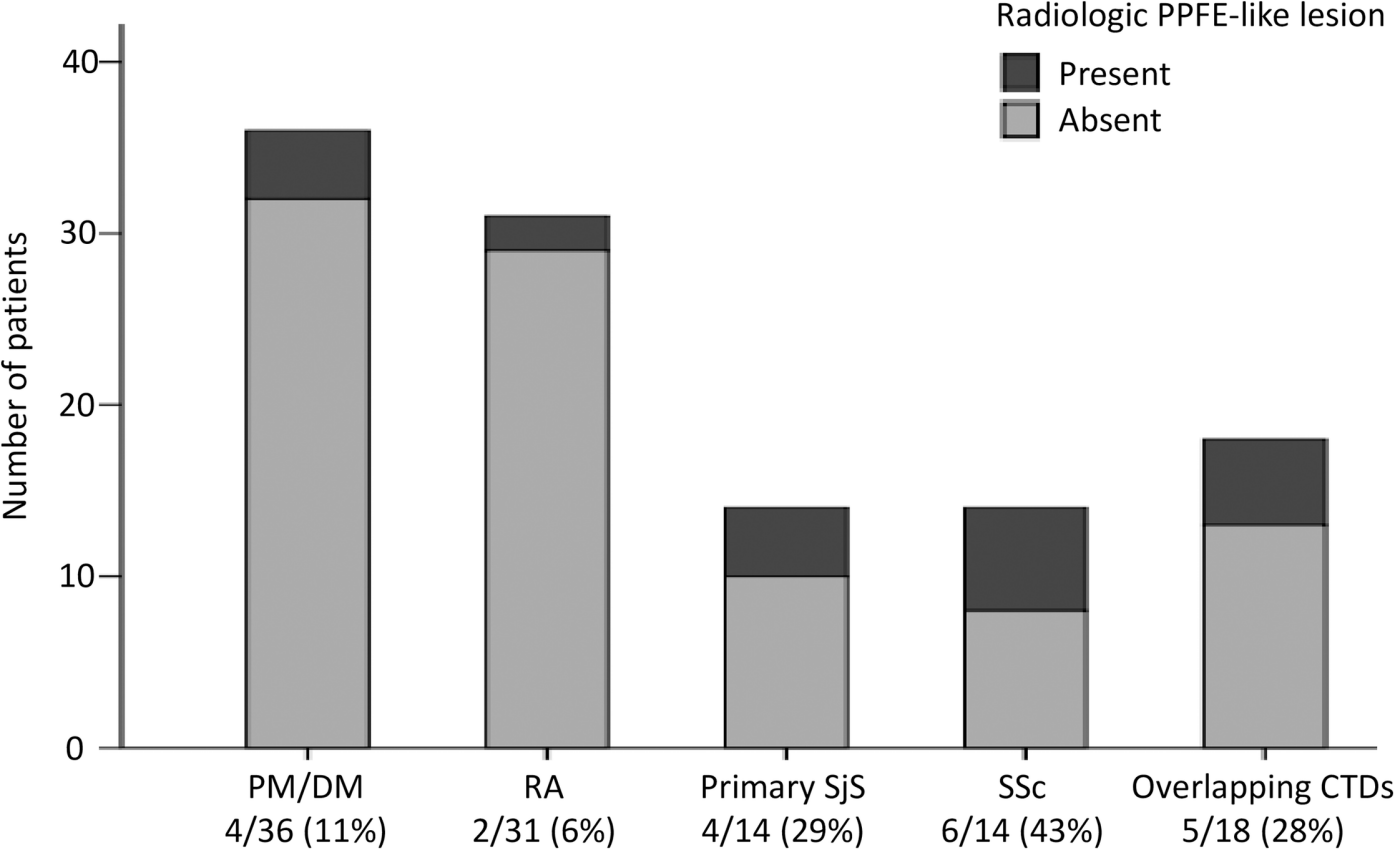
Prevalence of radiologic pleuroparenchymal fibroelastosis (PPFE)-like lesion in each connective tissue disease. Definition of abbreviations: CTD = connective tissue disease; DM = dermatomyositis; PM = polymyositis; RA = rheumatoid arthritis; SjS = Sjögren's syndrome; SSc = systemic sclerosis.

The baseline patient characteristics upon diagnosis of CTD-related ILD and comparison of patients based on the presence of radiologic PPFE-like lesion are summarized in Table 1. In the total cohort, the median age was 62 years (interquartile range: 56–70 years) and 47 patients (42%) were males. Radiologic PPFE-like lesion was found in 21 patients (19%) and were relatively frequent in those with systemic sclerosis (6/14: 43%) and primary Sjögren's syndrome (4/14: 29%) (Fig 3). No patient had a family history of ILD or a past history of pulmonary tuberculosis, radiotherapy to the lungs, cytotoxic chemotherapy, or tissue transplantation. Patients with PPFE-like lesion were significantly older and had lower body mass index than those without the lesion. There were no significant differences in PaO_2_, % predicted FVC, and % predicted DLCO between the groups, but the ratio of RV to TLC was significantly higher at a median value of 44% (interquartile range: 37%–47%) in patients with PPFE-like lesion.

**Table 1:** Clinical findings at the diagnosis of connective tissue disease-related interstitial lung disease.

		Radiologic PPFE-like lesion		
	Total (n = 113)	Absent (n = 92)	Present (n = 21)	*p*
Age (years)	62 (56, 70)	62 (55, 68)	63 (63, 74)	0.03
Male	47	40	7	0.47
Body mass index (kg/m^2^)	22.2 (20.2, 24.4) (n = 109)	22.4 (20.5, 24.8) (n = 88)	19.8 (17.3, 22.4)	<0.01
Current or former smoker	57	48	9	0.48
Smoking pack-year	0 (0, 38) (n = 111)	5 (0, 38)	0 (0, 40) (n = 19)	0.47
Occupational dust exposure	16 (n = 94)	11 (n = 76)	5 (n = 18)	0.18
Dyspnea	48 (n = 84)	39 (n = 66)	9 (n = 18)	0.59
Fine crackles on chest auscultation	93 (n = 106)	76 (n = 87)	17 (n = 19)	1.00
Clubbed finger	11 (n = 85)	8 (n = 70)	3 (n = 15)	0.40
PaO_2_ on room air (Torr)	77 (69, 88) (n = 99)	80 (69, 88) (n = 81)	74 (69, 91) (n = 18)	0.47
PaCO_2_ (Torr)	40 (38, 42) (n = 99)	39 (38, 42) (n = 81)	41 (39, 44) (n = 18)	0.16
LDH (IU/L)	246 (211, 317) (n = 109)	250 (214, 325) (n = 88)	239 (189, 302)	0.23
KL-6 (U/mL)	791 (520, 1185) (n = 109)	800 (534, 1123) (n = 88)	674 (491, 1361)	0.84
SP-D (ng/mL)	177 (106, 271) (n = 104)	173 (102, 262) (n = 83)	207 (118, 294)	0.46
% predicted FVC (%)	76 (64, 87) (n = 104)	77 (65, 89) (n = 84)	67 (60, 86) (n = 20)	0.15
% predicted DLCO (%)	71 (58, 94) (n = 48)	73 (60, 92) (n = 36)	67 (42, 97) (n = 12)	0.49
Ratio of RV to TLC (%) (reference range: 22%–40%)	39 (35, 44) (n = 47)	38 (33, 41) (n = 35)	44 (37, 47) (n = 12)	0.02
BALF-lymphocyte (%)	6 (4, 13) (n = 82)	6 (4, 14) (n = 67)	6 (4, 10) (n = 15)	0.59

Data are presented as n or median with interquartile range. All *p* values are evaluated by comparing between the patients with and without radiologic PPFE-like lesion, using Chi-square test, Fisher’s exact test, or Mann–Whitney’s U test as appropriate.

Definition of abbreviations: BALF = bronchoalveolar lavage fluid; DLCO = diffusing capacity of the lung for carbon monoxide; FVC = forced vital capacity; KL-6 = Krebs von den Lungen-6; LDH = lactate dehydrogenase; PPFE = pleuroparenchymal fibroelastosis; RV = residual volume; SP-D = surfactant protein-D; TLC = total lung capacity.

### Radiologic and pathologic findings

The radiologic findings at the time of CTD-related ILD diagnosis are shown in Table 2. More than half of the patients (12/21: 57%) with PPFE-like lesion were classified as UIP or possible UIP pattern, whereas the majority without this lesion showed an NSIP+OP pattern. Patients with PPFE-like lesion showed significantly more extensive reticular abnormalities and less ground-glass attenuation that were occasionally upper lobe-predominant.

**Table 2:** Radiologic and pathologic findings at the diagnosis of connective tissue disease-related interstitial lung disease.

	Radiologic PPFE-like lesion		*p*
	Absent	Present	
**Radiologic findings**	**n = 92**	**n = 21**	
Major pattern			0.01
UIP	6 (7)	5 (24)	
Possible UIP	25 (27)	7 (33)	
NSIP	5 (5)	0 (0)	
NSIP+OP	42 (46)	2 (10)	
OP	6 (7)	3 (14)	
Unclassifiable	8 (9)	4 (19)	
Cephalocaudal distribution			<0.01
Upper lobe-predominant	0 (0)	3 (14)	
Lower lobe-predominant	86 (93)	14 (67)	
Diffuse	6 (7)	4 (19)	
Extent of each finding (semi-quantitative score: 0/1/2/3/4)*			
Reticular abnormalities	54 (59)/26 (28)/12 (13)/0 (0)/0 (0)	5 (24)/10 (48)/6 (29)/0 (0)/0 (0)	<0.01
Ground-glass attenuation	10 (11)/72 (78)/8 (9)/2 (2)/0 (0)	9 (43)/10 (48)/2 (10)/0 (0)/0 (0)	0.01
Consolidation	45 (49)/42 (46)/5 (5)/0 (0)/0 (0)	12 (57)/8 (38)/1 (5)/0 (0)/0 (0)	0.51
Emphysema	62 (67)/27 (29)/3 (3)/0 (0)/0 (0)	17 (81)/3 (14)/1 (5)/0 (0)/0 (0)	0.26
**Pathologic findings**	**n = 33**	**n = 3**	
Major pattern			0.096
UIP	3 (9)	2 (67)	
NSIP	18 (55)	1 (33)	
OP	3 (9)	0 (0)	
LIP	4 (12)	0 (0)	
Others	5 (15)	0 (0)	
Severity of each finding (semi-quantitative score: 0/1/2/3)^†^			
Interstitial fibrosis	6 (18)/14 (42)/10 (30)/3 (9)	0 (0)/1 (33)/2 (67)/0 (0)	0.41
Fibroblastic foci	23 (70)/9 (27)/1 (3)/0 (0)	1 (33)/1 (33)/1 (33)/0 (0)	0.13
Lymphoplasmacytic infiltration	1 (3)/8 (24)/17 (52)/7 (21)	0 (0)/1 (33)/2 (67)/0 (0)	0.55
Lymphoid follicles with germinal center	12 (36)/7 (21)/4 (12)/10 (30)	2 (67)/0 (0)/1 (33)/0 (0)	0.32
Emphysema	29 (88)/2 (6)/2 (6)/0 (0)	3 (100)/0 (0)/0 (0)/0 (0)	0.53

Data are presented as n (%). All *p* values are evaluated by comparing between the patients with and without radiologic PPFE-like lesion, using Chi-square test or Mann–Whitney’s U test as appropriate.

* The scores are evaluated in whole lungs: 0 (absent), 1 (0%–25%), 2 (25%–50%), 3 (50%–75%), and 4 (> 75%).

^†^ 0 (absent), 1 (mild), 2 (moderate), and 3 (severe).

Definition of abbreviations: LIP = lymphocytic interstitial pneumonia; NSIP = nonspecific interstitial pneumonia; OP = organizing pneumonia; PPFE = pleuroparenchymal fibroelastosis; UIP = usual interstitial pneumonia.

**Fig 4:**
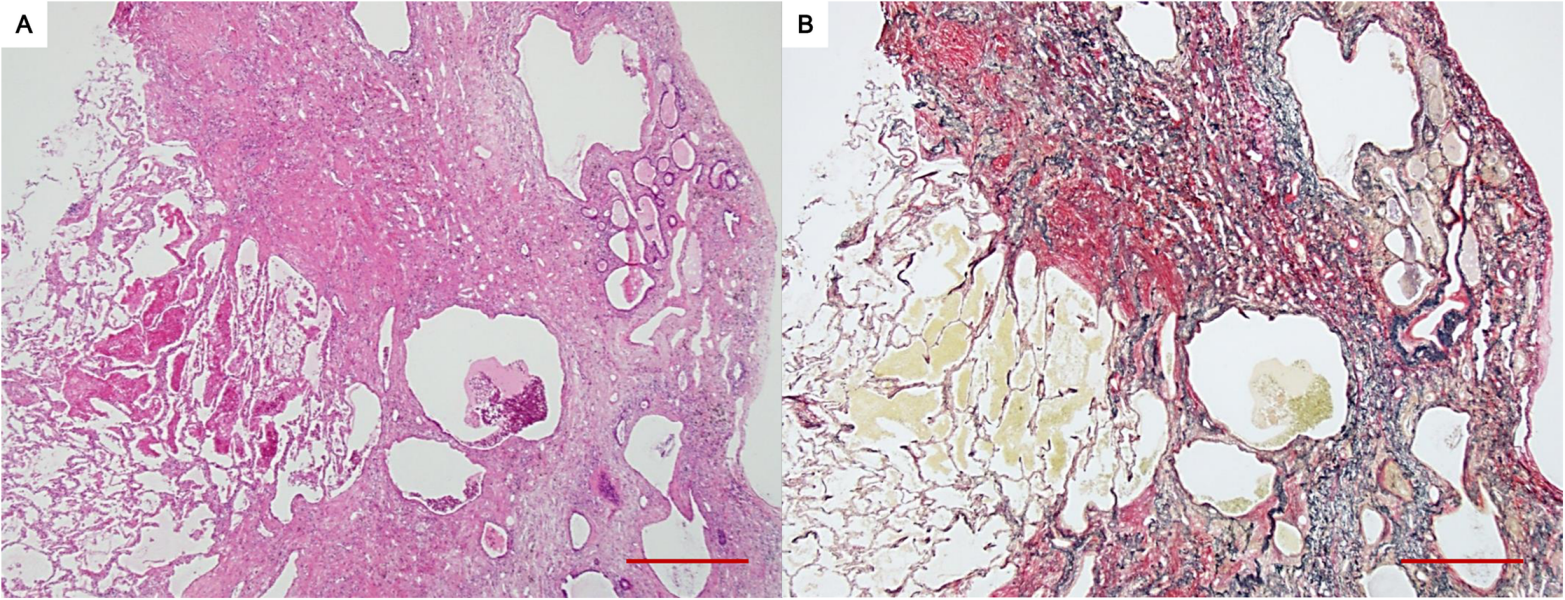
Pathologic images of a patient with primary Sjögren's syndrome and radiologic pleuroparenchymal fibroelastosis-like lesion (Patient 8 in S1 Table). Subpleural dense fibroelastosis adjacent to an almost normal lung parenchyma is shown. In the fibroelastic lesion, architectural distortion with multiple cystic changes is observed (A: hematoxylin–eosin; B: elastic van Gieson. Scale bar: 100 μm).

Table 2 also shows the comparison of surgical lung biopsy findings between 3 patients with radiologic PPFE-like lesion and 33 patients without the lesion. The former 3 patients were pathologically diagnosed as UIP (n = 2) and fibrotic NSIP (n = 1), whereas the latter 33 patients were commonly classified as cellular or fibrotic NSIP. There were no patients who showed a typical finding of pathologic PPFE on surgical lung biopsy specimens even in the upper lobes of patients with radiologic PPFE-like lesion. In contrast, the autopsy slides of a patient with radiologic PPFE-like lesion and primary Sjögren's syndrome, who did not receive surgical lung biopsy, revealed dense subpleural fibroelastosis on the upper lobe, which was characteristic of PPFE (Fig 4). The patient also showed UIP on the lower lobe.

### Clinical course and prognosis

The clinical course of the patients according to the presence or absence of radiologic PPFE-like lesion is compared on Table 3. The incidence of respiratory complications, such as acute exacerbation of ILD, respiratory tract infection, and lung cancer, did not differ between the groups. However, patients with radiologic PPFE-like lesion more frequently developed pneumothorax and/or pneumomediastinum than those without (38% vs. 15%, p = 0.03). Among the former patients, hypercapnia was frequently observed (5/8: 63%; S1 Table). Progression of the radiologic PPFE-like lesion was observed in 13 of 21 patients (62%) during observation (S1 Table).

**Table 3:** Comparison of the clinical course in connective tissue disease-related interstitial lung disease.

	Radiologic PPFE-like lesion		*p*
**Complications and treatment**	**n = 92**	**n = 21**	
Acute exacerbation of ILD	8 (9)	4 (19)	0.23
Respiratory tract infection	18 (20)	6 (29)	0.38
Lung cancer	6 (7)	0 (0)	0.59
Pneumothorax and/or pneumomediastinum	14 (15)	8 (38)	0.03
Long-term oxygen therapy	13 (14)	6 (29)	0.12
Immunosuppressive treatment	72 (78)	16 (76)	0.78
**Causes of death**	**n = 17**	**n = 6**	0.07
Worsening of chronic respiratory failure	7 (41)	3 (50)	
Acute exacerbation of ILD	0 (0)	2 (33)	
Respiratory tract infection	2 (12)	1 (17)	
Lung cancer	3 (18)	0 (0)	
Non-respiratory diseases	5 (29)	0 (0)	

Data are presented as n (%). All *p* values are evaluated by comparing between the patients with and without radiologic PPFE-like lesion, using Chi-square test or Fisher’s exact test as appropriate.

Definition of abbreviations: ILD = interstitial lung disease; PPFE = pleuroparenchymal fibroelastosis.

In 21 patients with radiologic PPFE-like lesion, the pulmonary function data one year (between 9 months and 15 months) after the diagnosis of CTD-related ILD were available in 16 patients. Interestingly, patients with “progressive” PPFE-like lesion (n = 10) commonly showed the deterioration of their pulmonary function (median change of % predicted FVC in one year: −7.6%; interquartile range: from −18.4% to +5.2%). In contrast, most patients with “stable” PPFE-like lesion (n = 6) maintained or improved their function (median change of % predicted FVC in one year: +11.4%; interquartile range: from +0.3% to +20.0%).

In the total cohort, the median follow-up period was 60.7 months (interquartile range: 31.0–99.2 months). During the period, there were 6 and 17 deaths, respectively, among patients with and without radiologic PPFE-like lesion. No patients received lung transplantation. The causes of death were variable in patients without radiologic PPFE-like lesion. On the other hand, in the six patients with the lesion, all the causes of death were respiratory diseases, such as worsening of chronic respiratory failure (n = 3), acute exacerbation of ILD (n = 2), and respiratory tract infection (n = 1). In 5 of the 6 non-surviving patients, the PPFE-like lesions were “progressive” (S1 Table).

**Fig 5:**
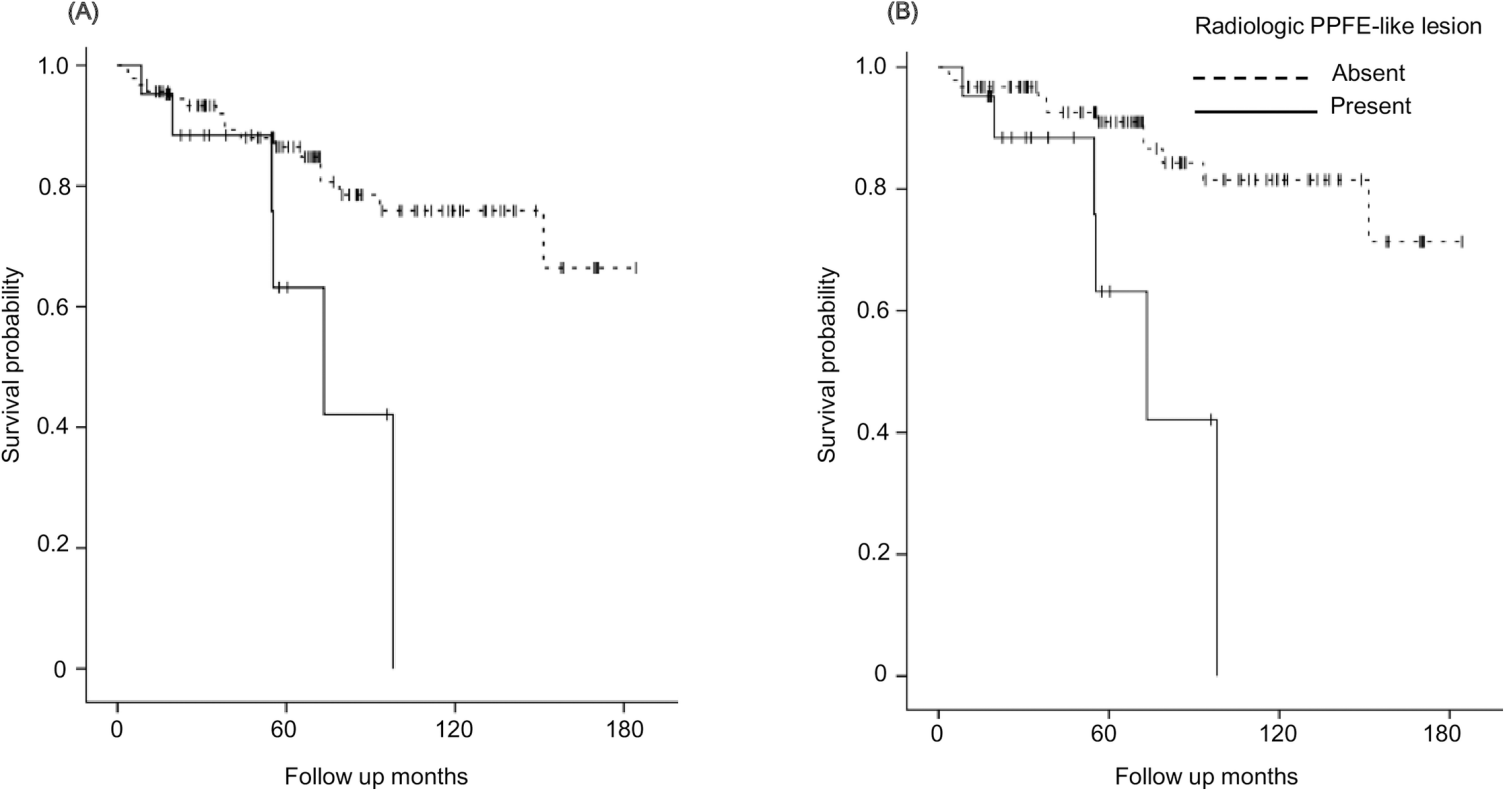
Comparison of survival curves between patients with and without radiologic pleuroparenchymal fibroelastosis (PPFE)-like lesion. The curves are drawn when the primary endpoint is set at death by all causes (A) and by respiratory diseases (B).

The survival curves are shown in Fig 5. The survival of patients with radiologic PPFE-like lesion was significantly worse than that of patients without the lesion (p = 0.02: Fig 5A); this trend was more evident when the patients who died of non-respiratory causes were classified as censored cases (p <0.01: Fig 5B). As shown in Table 4, our multivariate analysis identified the presence of radiologic PPFE-like lesion as a significant risk factor for death due to respiratory diseases (hazard ratio: 4.10, 95% confidence interval: 1.33–12.65, p = 0.01), which was independent of radiologic UIP compatibility.

**Table 4:** Analysis for the prognostic factors in connective tissue disease-related interstitial lung disease.

	Univariate analysis			Multivariate analysis		
Variables	HR	95% CI	*p*	HR	95% CI	*p*
**Death by all causes**						
Age	1.09	1.04–1.15	<0.01	1.09	1.03–1.15	<0.01
Male	1.73	0.76–3.97	0.19	–	–	–
% predicted FVC (%)	0.98	0.96–1.003	0.09	–	–	–
% predicted DLCO (%)	0.96	0.93–1.002	0.06	–	–	–
Radiologic UIP pattern	4.47	1.45–13.81	0.01	1.28	0.35–4.64	0.71
Radiologic PPFE-like lesion	3.06	1.17–8.00	0.02	2.44	0.86–6.91	0.09
**Death by respiratory diseases**						
Age	1.10	1.04–1.17	<0.01	1.10	1.03–1.17	0.01
Male	1.25	0.48–3.25	0.65	–	–	–
% predicted FVC (%)	0.99	0.96–1.02	0.45	–	–	–
% predicted DLCO (%)	0.97	0.93–1.01	0.15	–	–	–
Radiologic UIP pattern	4.71	1.27–17.46	0.02	1.00	0.23–4.38	1.00
Radiologic PPFE-like lesion	4.73	1.69–13.19	<0.01	4.10	1.33–12.65	0.01

All *p* values are evaluated by Cox’s proportional hazards regression model.

Definition of abbreviations: CI = confidence interval; DLCO = diffusing capacity of the lung for carbon monoxide; FVC = forced vital capacity; HR = hazard ratio; PPFE = pleuroparenchymal fibroelastosis; UIP = usual interstitial pneumonia.

## Discussion

In our cohort of CTD-rerated ILD, radiologic PPFE-like lesion was detected in as many as 19% of patients, most commonly in those with radiologic and/or pathologic UIP pattern. Intriguingly, despite the possibility that our criteria might have included incidental apical caps, the presence of PPFE-like lesion was identified as an independent poor prognostic factor. To the best of our knowledge, our result is the first to delineate the clinical significance of radiologic PPFE-like lesion in CTD-related ILD.

Pulmonary apical cap is not a rare radiologic finding and the prevalence in the general population was reported as around 10%, more predominantly in older individuals [1]. However, its prevalence in patients with CTD, regardless of an associated ILD, is unknown. Importantly, apical cap is principally a non-progressive condition. In our 21 patients with radiologic PPFE-like lesion, the progression of the lesions was not observed in 8 patients; some of the lesions might correspond to apical caps. The etiology of apical cap has been speculated to be a combination of repeated low-grade inflammation and chronic apical ischemia that leads to localized fibrosis [2]. Of note, the radiologic and pathologic features of apical cap occasionally overlap with those of PPFE and subpleural fibrosis due to UIP on the upper lobes [3,6]. In fact, although the number of patients with radiologic PPFE-like lesion who received pathologic evaluation was small, two of three patients had upper lobe UIP on surgical lung biopsy and another patient showed upper lobe PPFE on lung autopsy.

PPFE is a distinct pathologic pattern and the idiopathic type has been recently recognized as a rare form of IIPs [14,18,21,22]. In previous studies, PPFE of the upper lobe was found to coexist with other ILD patterns, particularly UIP on the lower lobes [5,14,23]. Oda et al. reported a PPFE histopathology of the upper lobes in nine IPF patients, who were emaciated, had increased ratio of RV to TLC, had high complication rate of pneumothorax and/or pneumomediastinum, and were in hypercapnic respiratory failure [23]. Most of these features were commonly observed in 21 of our CTD-related ILD patients with radiologic PPFE-like lesion. Additionally, Oda et al. showed that the prognosis of IPF with PPFE tended to be worse than that of IPF without PPFE, perhaps akin to our observation in patients with CTD-related ILD with radiologic PPFE-like lesion. These similarities between the data of Oda et al. and ours suggest that the majority of our patients with radiologic PPFE-like lesion but without pathologic evaluation might have pathologic PPFE, particularly in patients with “progressive” lesion.

In our CTD-related ILD patients, the presence of radiologic PPFE-like lesion was a significant risk factor for death due to respiratory diseases. One of the explanations may be the predominance of UIP pattern, which is a well-known poor prognostic determinant, particularly in IIPs and rheumatoid arthritis-related ILD [15,24]. Generally, patients with UIP have a high risk of acute exacerbation of ILD. Actually, among the patients with radiologic PPFE-like lesion in the present study, those who developed acute exacerbation of ILD (n = 4) exclusively exhibited radiologic or pathologic UIP pattern and the two of them died of the event. However, the prognostic effect of radiologic PPFE-like lesion remained significant even after adjusting for UIP compatibility in the multivariate analysis. On the other hand, our patients with radiologic PPFE-like lesion showed significantly more extensive reticular abnormalities on HRCT, which has also been suggested as a poor prognostic factor in various ILDs [25–28]. Therefore, it remained unclear whether the upper lobe lesion itself affected the clinical course or the lesion was merely a secondary change seen in cases with poor prognosis. Nevertheless, we think that the presence of radiologic PPFE-like lesion can be easily evaluated and is worth paying attention to, particularly when it is progressive.

The major limitation of the present study was that it was a small, single-center retrospective study. In particular, the small number of subjects who underwent pathologic evaluation made it difficult to fully evaluate the correlation between the radiologic and pathologic findings. The distinction between radiologic PPFE-like lesion with clinical significance and pure apical caps remains unclear, if at all possible. It may be helpful to confirm disease progression by serial radiologic assessment.

## Conclusion

Radiologic PPFE-like lesion, which may present as not only PPFE but also apical cap and upper lobe subpleural fibrosis commonly due to UIP, was not uncommon and was associated with worse prognosis in patients with CTD-related ILD.

## Supporting information

S1 Table Summary of patients with connective tissue disease-related interstitial lung disease and radiologic pleuroparenchymal fibroelastosis-like lesion. (DOCX)
