# Supplementary material for: Radiologic pleuroparenchymal fibroelastosis-like lesion in connective tissue disease-related interstitial lung disease
Source: PLoS One. 2017 Jun 30;12(6):e0180283. doi: 10.1371/journal.pone.0180283 (PMC5493376; doi:10.1371/journal.pone.0180283)
Supplement: S1 Table — (DOCX) [file pone.0180283.s001.docx]

**S1 Table. Summary of patients with connective tissue disease-related interstitial lung disease and radiologic PPFE-like lesion**

| Pt | CTD | Gender/age (years) | %FVC (%) | %DLCO (%) | HRCT pattern | Pathology (method) | Treatment | Progression of PPFE-like lesion | Hypercapnia | Respiratory complications | Outcome (cause of death) | Follow-up months |
| --- | --- | --- | --- | --- | --- | --- | --- | --- | --- | --- | --- | --- |
| 1 | PM/DM | F/63 | 86 | NE | NSIP+OP | NE | CS; IS | No | No | None | Alive | 57.4 |
| 2 | PM/DM | F/75 | 121 | 97 | Possible UIP | NE | None | No | No | None | Alive | 17.7 |
| 3 | PM/DM | M/72 | 91 | 58 | Possible UIP | NE | CS; IS | No | No | RTI | Alive | 18.7 |
| 4 | PM/DM | F/52 | 64 | 62 | OP | NE | CS; IS | Yes | Yes | P-M; RTI | Alive | 17.3 |
| 5 | RA | M/63 | 90 | NE | Unclassifiable | NE | CS | No | No | None | Alive | 95.9 |
| 6 | RA | M/77 | 69 | NE | Possible UIP | NE | CS | No | Yes | P-M; RTI | Dead (RTI) | 8.5 |
| 7 | SjS | F/71 | 45 | 72 | Possible UIP | NE | CS | Yes | No | None | Dead (CRF) | 55.3 |
| 8 | SjS | F/74 | 86 | 95 | UIP | Upper lobe PPFE with lower lobe UIP (autopsy) | CS | Yes | No | AE; P-M; RTI | Dead (AE) | 73.4 |
| 9 | SjS | F/42 | 73 | NE | Unclassifiable | UIP (SLB) | CS; IS; pirfenidone | Yes | Yes | P-M | Dead (CRF) | 98.0 |
| 10 | SjS | F/70 | 60 | NE | OP | UIP (SLB) | CS; IS | Yes | No | AE; P-T; P-M | Dead (AE) | 54.8 |
| 11 | SSc | F/63 | 66 | 110 | Unclassifiable | NE | None | Yes | Yes | None | Alive | 17.7 |
| 12 | SSc | F/62 | 65 | 42 | Possible UIP | NE | CS; IS | No | No | None | Alive | 32.5 |
| 13 | SSc | M/56 | 34 | 27 | NSIP+OP | NE | CS; IS; pirfenidone | Yes | No | AE | Alive | 60.4 |
| 14 | SSc | M/63 | NE | NE | UIP | NE | CS; IS | Yes | Yes | AE; RTI | Alive | 38.6 |
| 15 | SSc | M/63 | 86 | NE | Possible UIP | NE | CS; IS | Yes | Yes | P-T | Alive | 25.8 |
| 16 | SSc | M/78 | 85 | 148 | UIP | NE | None | Yes | No | None | Alive | 18.4 |
| 17 | SjS+SSc | F/74 | 68 | NE | OP | NE | None | Yes | No | P-T; P-M | Dead (CRF) | 19.6 |
| 18 | SjS+SSc | F/76 | 51 | NE | UIP | NE | None | Yes | Yes | P-T | Alive | 22.5 |
| 19 | RA+SjS+SSc | F/62 | 64 | 43 | Possible UIP | Fibrotic-NSIP with follicular bronchiolitis (SLB) | CS; IS | Yes | No | None | Alive | 30.8 |
| 20 | PM/DM+SjS+SSc+SLE | F/64 | 60 | 72 | UIP | NE | CS | No | No | RTI | Alive | 18.2 |
| 21 | PM/DM+SjS+SSc+SLE | F/63 | 56 | 31 | Unclassifiable | NE | CS | No | No | None | Alive | 47.6 |

Definition of abbreviations: AE = acute exacerbation; CRF = chronic respiratory failure; CS = corticosteroid; CTD = connective tissue disease; DLCO = diffusing capacity of the lung for carbon monoxide; DM = dermatomyositis; FVC = forced vital capacity; HRCT = high-resolution computed tomography; IS = immunosuppressant; NE = not evaluated; NSIP = nonspecific interstitial pneumonia; OP = organizing pneumonia; PM = polymyositis; P-M = pneumomediastinum; PPFE = pleuroparenchymal fibroelastosis; P-T = pneumothorax; RA = rheumatoid arthritis; RTI = respiratory tract infection; SjS = Sjögren's syndrome; SLB = surgical lung biopsy; SLE = systemic lupus erythematosus; SSc = systemic sclerosis; UIP = usual interstitial pneumonia.
